# Diagnostic Performance of ChatGPT-4o in Classifying Idiopathic Epiretinal Membrane Based on Optical Coherence Tomography

**DOI:** 10.3390/jcm15010292

**Published:** 2025-12-30

**Authors:** Tadanobu Sato, Taro Kuramoto

**Affiliations:** Department of Ophthalmology, Uonuma Kikan Hospital, Minamiuonuma 949-7302, Niigata, Japan; momotaro.1129.tk@gmail.com

**Keywords:** ChatGPT-4o, large language model, epiretinal membrane, Govetto classification, optical coherence tomography

## Abstract

**Background/Objectives:** Recent advances in large language models (LLMs) have enabled the multimodal interpretation of medical images, but their agreement in ophthalmology issues remains underexplored. This study evaluated the ability of ChatGPT-4o, a multimodal LLM, to classify idiopathic epiretinal membrane (ERM) using optical coherence tomography (OCT) based on the Govetto classification. **Methods:** This retrospective study included 250 eyes of 250 patients with idiopathic ERM who visited Uonuma Kikan Hospital between June 2015 and April 2025. Horizontal B-scan OCT images were independently classified into four stages by two masked ophthalmologists; cases with disagreement were excluded. ChatGPT-4o was prompted to identify ocular diseases and classify ERM stage. Agreement between ChatGPT-4o and ophthalmologists was evaluated using weighted Cohen’s κ, and logistic regression identified factors associated with disagreement. **Results:** Among 272 eligible eyes, 250 were analyzed (Stage 1: 87; Stage 2: 76; Stage 3: 63; Stage 4: 24). ChatGPT-4o identified the presence of ERM in 26.4% of cases on the first prompt. The perfect agreement rate for Govetto staging was 46.0%, with a weighted κ of 0.513 (95% CI: 0.420–0.605; *p* < 0.001), indicating moderate agreement. Disagreement was significantly associated with the presence of ectopic inner foveal layer (EIFL) (OR = 0.528, 95% CI: 0.312–0.893; *p* = 0.017). **Conclusions:** ChatGPT-4o showed moderate agreement with ophthalmologists in Govetto classification of idiopathic ERM using OCT images. Although its agreement was limited, the model demonstrated partial ability to recognize retinal structures, providing insight into the current capabilities and limitations of multimodal large language models in ophthalmic image interpretation.

## 1. Introduction

Chat Generative Pre-trained Transformer (ChatGPT), developed by OpenAI, is a large language model (LLM) capable of understanding and generating human-like text. It was initially released in November 2022. It is based on a large language model architecture employing neural-network machine learning with tens of billions of parameters, enabling advanced natural language processing and human-like text summarization. The first widely available version utilized GPT-3.5, comprising 175 billion parameters and offered free of charge to all users. In March 2023, OpenAI introduced GPT-4, which was further enhanced in September 2023 with the capability to process visual inputs. The most recent advancement, GPT-4o, launched in May 2024, extended the model’s capabilities to encompass multimodal input and output, including text, images, and audio [1]. Recent advancements, including multimodal input capability in ChatGPT-4o, have enabled the model to interpret medical images in addition to text, expanding its potential utility in clinical and diagnostic settings. It has been demonstrated that ChatGPT was able to achieve the passing threshold of almost 60% accuracy on the United States Medical Licensing Exam without specialized input from human trainers and ChatGPT-4 provides accurate answers to text-based questions regarding retinal diseases [2,3]. ChatGPT appeared capable of responding to long user-written eye health posts and largely generated appropriate responses that did not differ significantly from ophthalmologist-written responses in terms of incorrect information, likelihood of harm, extent of harm, or deviation from ophthalmologist community standards [4]. It is unclear how widely ChatGPT is used in clinical practice within the field of ophthalmology, but rate of using ChatGPT are likely advancing. However, its performance in image-based classification tasks, particularly within ophthalmology, remains underexplored.

**Figure 1:**
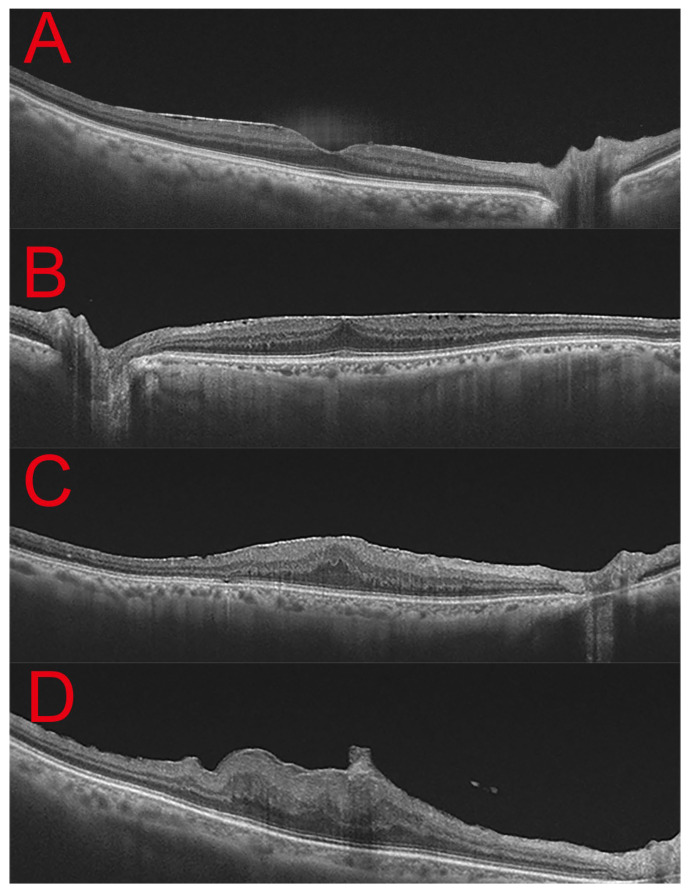
Representative spectral-domain OCT images of idiopathic epiretinal membrane according to the Govetto classification (Stages 1–4). (**A**) **Stage 1:** Preserved foveal depression with a thin epiretinal membrane (ERM) and minimal distortion of the inner retinal layers. (**B**) **Stage 2:** Loss of the foveal depression with a continuous but thickened inner retinal layer, without ectopic inner foveal layers (EIFL). (**C**) **Stage 3:** Presence of a continuous EIFL bridging the foveal area, causing flattening of the foveal contour. (**D**) **Stage 4:** ERM with severe retinal distortion, thickened EIFL, and loss of layered retinal architecture.

Epiretinal membrane (ERM) is a common macular pathology that can be systematically staged using optical coherence tomography (OCT) based on Govetto classification (Figure 1), which incorporates the presence of ectopic inner foveal layers (EIFL), central foveal contour changes, and outer retinal disruption [5]. Accurate staging is essential for assessing disease severity and determining appropriate surgical intervention.

Unlike prior studies such as the Taiwanese medical licensing exam evaluation using GPT-4/GPT-4o, which excluded image-based questions, our study uniquely investigates GPT-4o’s capability to directly interpret retinal OCT images for Govetto classification of idiopathic ERM [1]. Given that GPT-4o is fundamentally a language model with limited visual reasoning capability, we aimed to evaluate whether it can nonetheless approximate expert-level image-based classification performance. This study could provide new insights into the future integration of LLMs into ophthalmic workflows, particularly for preliminary image triage or decision support in resource-limited settings.

## 2. Materials and Methods

This retrospective, observational study was approved by the Research Ethics Committee of Uonuma Kikan Hospital (IRB No. E2025000401) and adhered to the tenets of the Declaration of Helsinki. Informed Consent Statement: Patient consent was waived due to the retrospective nature of the study, and an opt-out approach was employed in accordance with institutional guidelines. Information regarding the study was publicly disclosed, and patients were given the opportunity to decline participation. No patient opted out. We identified 272 eyes of 272 patients who visited Uonuma Kikan Hospital between June 2015 and April 2025 and were diagnosed with idiopathic ERM.

The exclusion criteria were as follows: secondary ERM due to retinal tear, uveitis, or prior vitreoretinal surgery; coexisting macular diseases such as lamellar macular hole (LMH), diabetic macular edema (DME), age-related macular degeneration, or retinal vein occlusion; and poor-quality OCT images that precluded accurate evaluation. All patients underwent a comprehensive ophthalmic examination, including best-corrected visual acuity (BCVA), slit-lamp biomicroscopy, dilated fundus examination, and swept-source optical coherence tomography (SS-OCT; Deep-Range Imaging [DRI] Triton, Topcon, Tokyo, Japan). All OCT images were acquired using the same SS-OCT device throughout the study period (2015–2025), ensuring consistency in image acquisition protocols. Twelve radial B-scan images centered on the fovea were acquired. Speckle noise was reduced by averaging 16 B-scans to improve image quality. To minimize the potential influence of image resolution, noise, and contrast on the analysis, only OCT images with an image quality score of 70 or higher, as provided by the device software, were included in this study. Images with significant signal attenuation or poor visualization of retinal layers, such as those caused by media opacity including advanced cataract, were excluded from the analysis. Horizontal B-scan OCT images through the fovea were used to classify idiopathic ERM according to Govetto classification system. Classification was performed independently by two masked ophthalmologists (Figure 1A–D), and cases with discrepancies were excluded. In addition to ERM staging, we assessed central foveal thickness (CFT), the thickness of the ectopic inner foveal layer (EIFL), and the integrity of the external limiting membrane (ELM), ellipsoid zone (EZ), and cotton ball sign (CBS) within a 1 mm radius centered on the fovea to evaluate the structural impact of ERM on the retina. CFT was automatically calculated using OCT within a 1 mm central foveal diameter range. The presence or absence of other OCT parameters was determined blindly by each ophthalmologist, and eyes with discrepancies were excluded from the study. The thickness of EIFL was manually measured tracing a straight line from the upper limit of the outer nuclear layer to the internal limiting membrane by two independent ophthalmologists. To assess inter-rater reliability, the intraclass correlation coefficient (ICC) was calculated between the two graders. The average of the two measurements was used for subsequent analyses. For each patient, the most recent OCT images and visual acuity results obtained at the time of their most recent visit were included in the study. For patients who underwent surgery for ERM, the OCT images and visual acuity obtained immediately prior to surgery were used in the study.

All ChatGPT-4o (OpenAI, San Francisco, CA, USA) interactions were conducted between May and June 2025 using the same model version. The memory function was disabled prior to each session to prevent cross-case information retention. Each OCT image was uploaded once and evaluated using a fixed two-step prompt without iterative refinement, feedback, or correction. This standardized protocol was adopted to enhance reproducibility. The procedure using ChatGPT-4o was as follows. The memory function was disabled prior to all interactions. For all cases, we first attached the OCT image and sent it with the following initial prompt: “What ocular diseases can be identified in this image?” Regardless of whether ERM was mentioned in the response, the second prompt was: “There is an epiretinal membrane. Please classify the ERM using the Govetto classification and select only one stage (Stage 1 to 4) that best fits this case.” The OCT image and text-based interaction were input manually only once, and the model’s classification and rationale were recorded for analysis. No additional clarification or prompting was provided during the session. For the initial recognition analysis, a response was considered correct if ERM was explicitly mentioned anywhere in the model’s output, including the section listing possible diagnoses, regardless of whether it was presented as the primary or most likely diagnosis.

Artificial Intelligence Use Statement: ChatGPT-4o was used as the artificial intelligence model evaluated in this study. The model was applied exclusively for image-based classification tasks as described in the Materials and Methods section. No automated data analysis, statistical processing, or manuscript writing was performed by the AI system.

All continuous variables are represented as mean ± standard deviation (SD) values. The Shapiro–Wilk test was performed to assess whether continuous variables were normally distributed. Differences in age, CFT and logMAR at stage1 to 4 of Govetto classification were determined using the Kruskal–Wallis test. Differences in ELM disruption, EZ disruption and CBS between each stage were analyzed using Fisher’s exact test. The initial recognition rate of ERM using ChatGPT was calculated. Agreement between ChatGPT and ophthalmologists was assessed as the perfect agreement rate of Govetto classification between ChatGPT-4o and ophthalmologists, further assessed using the weighted Cohen’s kappa coefficient, interpreted according to established criteria (<0.20, slight agreement; 0.21–0.40, fair agreement; 0.41–0.60, moderate agreement; 0.61–0.80, substantial agreement; >0.80, almost perfect agreement). A heatmap illustrating the confusion matrix between ChatGPT-4o and ophthalmologists was generated using the ggplot2 package in R (version 4.5.1). Logistic regression analysis was performed to identify factors associated with agreement and disagreement between ophthalmologists and ChatGPT-4o. Statistical analyses were primarily performed using SPSS Statistics (version 31.0.0.0; IBM Corp., Armonk, NY, USA). A *p*-value < 0.05 was considered statistically significant.

## 3. Results

**Figure 2:**
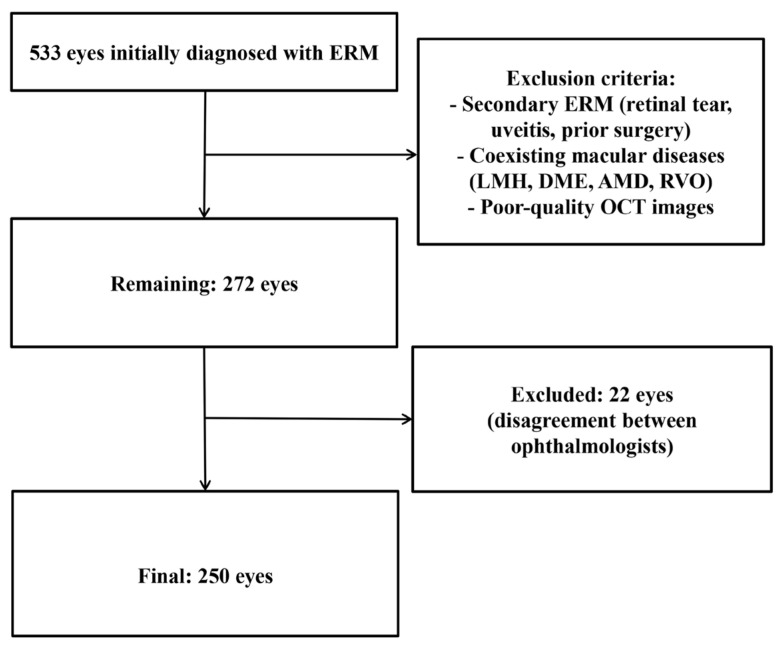
Flowchart of Patient Selection for the Study. Flow diagram illustrating the selection process of eyes included in this study. A total of 533 eyes initially diagnosed with idiopathic epiretinal membrane (ERM) were reviewed. After applying exclusion criteria—including secondary ERM (e.g., retinal tear, uveitis, prior ocular surgery), coexisting macular diseases (lamellar macular hole, diabetic macular edema, age-related macular degeneration, retinal vein occlusion), and poor-quality OCT images—272 eyes remained eligible for further assessment. Among them, 22 eyes were excluded due to disagreement in classification between ophthalmologists. Finally, 250 eyes were included in the analysis.

A total of 533 eyes were initially diagnosed with ERM. Eyes with LMH, DME, macular pucker secondary to other causes, or other exclusion criteria were removed, leaving 272 eyes. Of these, 22 eyes showing disagreement in Govetto classification or OCT parameters between the two ophthalmologists were excluded. The remaining 250 eyes from 250 patients with idiopathic ERM were included in the final analysis (Figure 2).

Table 1 shows the characteristics of patients classified according to the Govetto classification used by ophthalmologists. The distribution of stages was: Stage 1, n = 87; Stage 2, n = 76; Stage 3, n = 63; Stage 4, n = 24. Both logMAR and CFT increased progressively from stage 1 to stage 4, with statistically significant differences among the groups. A significant association was also found between disease stage and the presence of EZ disruption. ICC(2,2) for the thickness of the EIFL between the two ophthalmologists was 0.98 (95% CI: 0.97–0.99).

**Figure 3:**
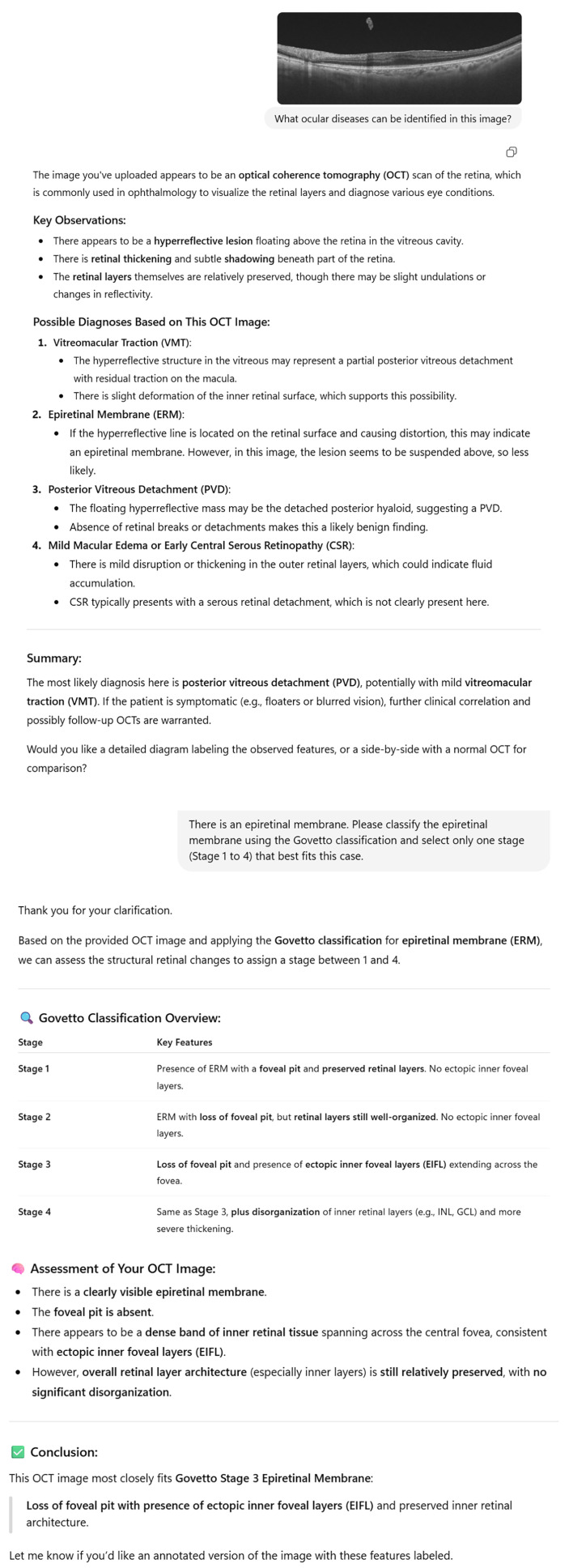
Example of ChatGPT-4o’s Govetto Classification (Stage 3) Based on an OCT Image. Representative optical coherence tomography (OCT) image uploaded to ChatGPT-4o, in which the model identified an epiretinal membrane (ERM) and classified the case as Govetto Stage 3. ChatGPT-4o’s assessment highlights the loss of the foveal pit and the presence of ectopic inner foveal layers (EIFL) spanning across the central macula, while noting relatively preserved inner retinal architecture without severe disorganization. These features meet the criteria of Stage 3 in the Govetto classification system. The structured output generated by ChatGPT-4o, including key observations, differential diagnoses, and classification rationale, is shown.

**Figure 4:**
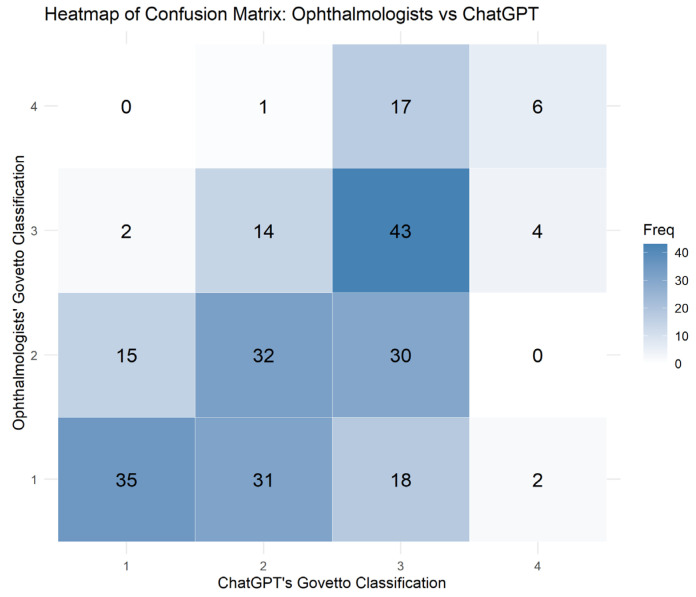
Heatmap of the Confusion Matrix Between Ophthalmologists and ChatGPT-4o for Govetto Classification. Heatmap visualization of the confusion matrix comparing Govetto classification between ophthalmologists and ChatGPT-4o across 250 OCT images with idiopathic epiretinal membrane. Each cell indicates the frequency of cases assigned to each combination of stages (1–4) by clinicians (vertical axis) and ChatGPT-4o (horizontal axis). Darker shading represents higher agreement frequencies. The diagonal cells represent correct agreement between ophthalmologists and ChatGPT-4o, with the highest concordance observed in Stage 3. Off-diagonal values illustrate misclassification patterns, highlighting specific stages where discrepancies were more common.

Figure 3 shows an example of the OCT image and the corresponding text-based interaction with ChatGPT-4o, illustrating the model’s reasoning process during classification. The initial recognition rate of ERM by ChatGPT-4o, based on an open-ended identification prompt, was 66 eyes (26.4%). In this study, the explanation of the Govetto classification in ChatGPT-4o’s response to the second question was accurate in all cases. Figure 4 presents a heatmap of the confusion matrix showing the distribution of Govetto classification results between ChatGPT-4o and ophthalmologists. While ChatGPT-4o tended to assign classifications close to expert judgments, a substantial number of misclassifications occurred between adjacent stages. Notably, the highest frequency was observed at the (ophthalmologists = 3, ChatGPT = 3) cell (n = 43), indicating strong concordance at this stage. However, under-classifications (e.g., ophthalmologists = 4, ChatGPT = 3) and over-classifications (e.g., ophthalmologists = 2, ChatGPT = 3) were also present.

The perfect agreement rate of Govetto classification between ChatGPT-4o and ophthalmologists was 46.0%. The weighted Cohen’s κ between ChatGPT-4o and the consensus grading by ophthalmologists was 0.513 (standard error = 0.047, 95% CI: 0.420–0.605; *p* < 0.001), indicating moderate agreement.

Table 2 summarizes the association between OCT-derived structural parameters and the agreement rate of Govetto classification between ChatGPT-4o and ophthalmologists. Among the evaluated parameters, the presence of an ectopic inner foveal layer (EIFL) was significantly associated with a lower likelihood of disagreement (*p* = 0.017). Other parameters, including the presence of a foveal pit, ELM disruption, EZ disruption and CBS showed no significant relationship with the agreement rate. Logistic regression analysis using the forward stepwise method, with disagreement as the dependent variable (agreement = 0, disagreement = 1), revealed that only the presence of EIFL was associated with reduced odds of disagreement (OR = 0.528, 95% CI: 0.312–0.893; *p* = 0.017) (Table 3). Other candidate variables, including logMAR, the presence of a foveal pit, ELM and EZ disruption, CBS, CFT, sex and patient age, were excluded from the final model.

**Table 3:** Logistic regression analysis of factors associated with agreement and disagreement between ChatGPT-4o and ophthalmologists in Govetto classification.

Variable	B	SE	*p*	OR (Exp(B))	95% CI for OR (Lower-Upper)	
Presence of EIFL	−0.639	0.269	0.017	0.528	0.312	0.893

B: regression coefficient; SE: standard error; OR: odds ratio; CI: confidence interval; EIFL: ectopic inner foveal layer. Logistic regression analysis of factors associated with agreement between ChatGPT-4o and ophthalmologists in Govetto classification. Agreement was coded as the dependent variable (agreement = 0, disagreement = 1).

## 4. Discussion

ChatGPT-4o demonstrates progress in contextual understanding and reasoning capabilities within medicine. However, no detailed reports exist on its imaging agreement for epiretinal membrane—a retinal disease—in ophthalmology. In this study, stage 4 cases are fewer in number and have poorer visual acuity compared to stages 1, 2, and 3, suggesting that the population is comparable to that reported in previous studies [5,6]. This study shows that significant differences in logMAR, CFT and EZ disruption were observed between groups in the Govetto classification. Doguizi et al. reported that as the Govetto classification increases, it is associated with decreased BCVA and increased central retinal thickness [6]. The population of this study was similar to that of previously reported results, making it considered appropriate as a research subject.

In this study, the initial idiopathic ERM diagnosis accuracy of ChatGPT-4o on the first question was 26.4%, indicating a low value. However, ChatGPT-4o’s responses to the second question provided accurate explanations of the Govetto classification in all cases, and the weighted Cohen’s kappa between ChatGPT-4o and the ophthalmologists grading was relatively good. Although the weighted Cohen’s κ value of 0.513 indicates moderate agreement between ChatGPT-4o and ophthalmologists, this level of concordance is insufficient for standalone clinical diagnostic use. In ophthalmic imaging, artificial intelligence systems intended for diagnostic purposes generally require substantial to near-perfect agreement to ensure clinical safety and reliability. Accordingly, the present findings should not be interpreted as evidence of clinical diagnostic capability. Rather, this study represents an exploratory evaluation of agreement between a general-purpose multimodal large language model and expert graders. At its current performance level, potential applications of ChatGPT-4o are limited to non-diagnostic contexts, such as educational use, demonstrative purposes, or hypothesis generation. The heatmap of the confusion matrix (Figure 4) further illustrates the distribution of agreement and misclassifications between ChatGPT-4o and expert graders. The highest concordance was observed for Stage 3, with 43 cases correctly classified by ophthalmologists and ChatGPT-4o, highlighting that the model performs best when morphological features such as EIFL are more pronounced. However, frequent misclassifications occurred between adjacent stages, most notably between Stage 2 and Stage 3, and between Stage 1 and Stage 3. This pattern indicates that ChatGPT-4o tends to overestimate the disease severity, especially by classifying milder stages (Stage 1–2) as more advanced (Stage 3). Momenaei et al. reported that the majority of ChatGPT4′s responses to questions about ERM were appropriate [3]. Based on these results, while it is possible to accurately guide users to existing information such as “appropriate” available online, in the context of image diagnosis, questions with restricted conditions may be necessary to improve the accuracy of responses. Mikhail et al. reported that recent systematic reviews and meta-analyses have demonstrated that artificial intelligence (AI) models can achieve high diagnostic performance in detecting ERM. For instance, a pooled analysis of 19 studies reported an overall accuracy of 93.8%, with sensitivity and specificity of 90.1% and 95.7%, respectively, across various machine learning and deep learning approaches, primarily using color fundus photographs or OCT scans as input modalities [7]. These results highlight the potential of conventional AI models, typically convolutional neural networks or vision transformer architectures, in screening and diagnostic settings. However, such models generally require large annotated datasets for supervised training [8]. and their applicability across institutions is limited by variations in imaging protocols and insufficient external validation.

In contrast, LLMs such as ChatGPT operate on fundamentally different principles [1]. Although GPT-4o has demonstrated strong performance in multimodal tasks, recent analyses indicate that its contextual reasoning and domain-specific knowledge integration remain limited [9]. These findings suggest that while the model can recognize visual features, its ability to apply clinical reasoning—such as stage classification based on structural OCT features—requires further investigation. While pretrained vision-based models are optimized for image recognition tasks, LLMs leverage vast pretrained knowledge bases and few-shot learning capabilities, enabling them to adapt to novel classification tasks without retraining. Our findings therefore extend the existing literature by exploring whether a general-purpose pretrained LLM can provide consistent staging comparable to ophthalmologists, a task not yet systematically assessed in previous AI studies. Patients frequently use the internet as a convenient source of health information and guidance, [10]. and the ability of conversational LLMs such as ChatGPT-4o to provide understandable, image-based feedback may facilitate patient engagement and education. This feature may allow its application in preliminary triage or as an educational aid. It is known that larger Govetto classification stages and the presence of EIFL correlate with poorer vision, [6,11]. and patients themselves may be able to easily assess the impact of ERM on vision by utilizing ChatGPT-4o.

Logistic regression revealed that the presence of EIFL was associated with reduced disagreement, likely because clearly identifiable EIFL serves as a salient morphological feature guiding Stage 3 classification by both ChatGPT-4o and ophthalmologists. Given that EIFL is central to the Govetto classification system, the further refinement of LLMs to enhance recognition of intraretinal layer alterations is warranted. Massraf et al. reported that the clinician-generated leaflet received the highest evaluation in the prompt of “Make a patient information leaflet on Descemet Membrane Endothelial Keratoplasty (DMEK) surgery.” Among LLM models, Claude 3.7 Sonnet scored the highest, followed by ChatGPT-4o [12]. Rayhan et al. evaluated Gemini and Copilot showed better reliability and quality about questions concerning keratoconus [13]. Thompson et al. found that ChatSonic provided the most reliable and comprehensible content regarding cataract surgery among several LLMs [14]. This study focused solely on ChatGPT-4o for LLM analysis and did not include comparisons with other LLMs. Had other LLMs been used, higher agreement rates in the Govetto classification may have been achieved. The analysis was limited to idiopathic ERM, enabling evaluation of how the presence or absence of EIFL affected Govetto staging by ChatGPT-4o and ophthalmologists. It was reported that EIFL becomes thinner in diabetic ERM. Therefore, ChatGPT-4o’s classification may be affected by patient history, such as the presence or absence of diabetes [15]. Due to the nature of LLMs, gradually training them on disease images is difficult, so there is hope for building an LLM that can more accurately grasp the features of images from existing reports.

Several limitations should be acknowledged. First, the number of stage 4 cases was relatively small, which may have limited statistical power for evaluating advanced disease. Second, this study was a single-center retrospective study. This single-center retrospective design using homogeneous OCT equipment limits external generalizability. A multicenter prospective study examining the diagnostic accuracy of LLMs would likely yield more precise information on its diagnostic capabilities. Third, because the model’s output is prompt-dependent, the results can vary significantly depending on the prompt content. In other words, it is conceivable that providing a more suitable prompt could potentially improve agreement. However, systematic prompt optimization or comparison of alternative protocol-based question structures was beyond the scope of this study, which aimed to evaluate agreement under a fixed and standardized interaction setting. Finally, this study excluded borderline cases with inter-observer disagreement, which may have resulted in an overestimation of agreement by preferentially retaining easier cases.

Despite these limitations, our findings provide the first evidence that a large language model with multimodal capabilities can approximate expert-level classification of ERM, suggesting a future role in teleophthalmology, clinical decision support, and automated reporting systems.

Future research should focus on integrating multimodal AI systems capable of combining OCT images with clinical parameters such as visual acuity and surgical outcomes. Such integration could improve both diagnostic accuracy and clinical applicability. Moreover, explainability and transparency in AI decision-making remain crucial for safe clinical deployment. As technology advances, large language models may serve not only as diagnostic assistants but also as communicative interfaces, automatically generating patient-friendly explanations or preliminary clinical notes from imaging data. Overall, this study highlights both the potential and the current limitations of agreement between a general-purpose multimodal LLM and expert graders when interpreting OCT images of idiopathic ERM.

## 5. Conclusions

This study demonstrated that ChatGPT-4o, a large multimodal language model, achieved moderate agreement with ophthalmologists in the Govetto classification of idiopathic epiretinal membrane based solely on OCT images. Although its initial diagnostic accuracy was limited, the model showed the ability to correctly recognize and describe structural retinal features, particularly in cases with more distinct morphological changes such as EIFL. However, these findings should not be interpreted as supporting direct clinical application, but as highlighting the current capabilities and limitations of multimodal large language models in ophthalmic image interpretation.

## Figures and Tables

**Table 1:** Characteristics of patients with idiopathic epiretinal membrane classified according to the Govetto classification by ophthalmologists.

	Stage 1	Stage 2	Stage 3	Stage 4	*p* Value
Patients, n	87	76	63	24	
Age, year (range)	73.6 ± 8.6 (52–94)	73.3 ± 9.2 (51–97)	73.8 ± 9.1 (55–92)	71.6 ± 5.5 (62–81)	^a^ 0.078
Central foveal thickness, μm	300.5 ± 45.0	350.3 ± 59.7	410.7 ± 60.4	521.1 ± 36.6	^a^ < 0.001 *
Ectopic inner foveal layer thickness, μm			130.8 ± 56.6		
External limiting membrane disruption, (%)	2/87 (2.3)	3/76 (3.9)	2/63 (3.2)	3/24 (12.5)	^b^ 0.192
Ellipsoid zone disruption, (%)	3/87 (3.4)	2/76 (2.6)	2/63 (3.2)	8/24 (33.3)	^b^ < 0.001 *
Cotton ball sign, (%)	4/87 (4.6)	8/76 (10.5)	5/63 (7.9)	2/24 (8.3)	^b^ 0.544
VA, logMAR	0.02 ± 0.12	0.06 ± 0.17	0.15 ± 0.21	0.44 ± 0.24	^a^ < 0.001 *

^a^ Kruskal–Wallis test; ^b^ χ^2^ test; * *p* < 0.05.

**Table 2:** Association between OCT structural parameters and agreement rate of Govetto classification between ChatGPT-4o and ophthalmologists.

		Govetto Classification		*p* Value
		Match (n)	Mismatch (n)	
Foveal pit	presence	35	48	0.392
	absence	80	87	
ELM disturbance	presence	6	4	0.279
	absence	109	131	
EZ disturbance	presence	7	8	0.957
	absence	108	127	
EIFL	presence	49	38	0.017 *
	absence	66	97	
Cotton ball sign	presence	10	9	0.546
	absence	105	126	

χ^2^ test, * *p* < 0.05. ELM: external limiting membrane; EZ: ellipsoid zone; EIFL: ectopic inner foveal layer.

## Data Availability

The data presented in this study are available on reasonable request from the corresponding author. The data are not publicly available due to privacy and ethical restrictions.

## References

[1] Wu Y.C., Wu Y.C., Chang Y.C., Yu C.Y., Wu C.L., Sung W.W. (2025). Advancing medical AI: GPT-4 and GPT-4o surpass GPT-3.5 in Taiwanese medical licensing exams. PLoS ONE.

[2] Kung T.H., Cheatham M., Medenilla A., Sillos C., De Leon L., Elepaño C., Madriaga M., Aggabao R., Diaz-Candido G., Maningo J. (2023). Performance of ChatGPT on USMLE: Potential for AI-assisted medical education using large language models. PLoS Digit. Health.

[3] Momenaei B., Wakabayashi T., Shahlaee A., Durrani A.F., Pandit S.A., Wang K., Mansour H.A., Abishek R.M., Xu D., Sridhar J. (2023). Appropriateness and readability of ChatGPT-4-generated responses for surgical treatment of retinal diseases. Ophthalmol. Retin..

[4] Bernstein I.A., Zhang Y., Govil D., Majid I., Chang R.T., Sun Y., Shue A., Chou J.C., Schehlein E., Christopher K.L. (2023). Comparison of ophthalmologist and large language model chatbot responses to online patient eye care questions. JAMA Netw. Open.

[5] Govetto A., Lalan R.A., Sarraf D., Figueroa M.S., Hubschman J.P. (2017). Insights into epiretinal membranes: Presence of ectopic inner foveal layers and a new optical coherence tomography staging scheme. Am. J. Ophthalmol..

[6] Doguizi S., Sekeroglu M.A., Ozkoyuncu D., Omay A.E., Yilmazbas P. (2018). Clinical significance of ectopic inner foveal layers in patients with idiopathic epiretinal membranes. Eye.

[7] Mikhail D., Gao A., Farah A., Mihalache A., Milad D., Antaki F., Popovic M.M., Shor R., Duval R., Kertes P.J. (2025). Performance of artificial intelligence-based models for epiretinal membrane diagnosis: A systematic review and meta-analysis. Am. J. Ophthalmol..

[8] De Fauw J., Ledsam J.R., Romera-Paredes B., Nikolov S., Tomasev N., Blackwell S., Askham H., Glorot X., O’donoghue B., Visentin D. (2018). Clinically applicable deep learning for diagnosis and referral in retinal disease. Nat. Med..

[9] Li N., Zhang J., Cui J. (2025). Have we unified image generation and understanding yet? An empirical study of GPT-4o’s image generation ability. arXiv.

[10] Calixte R., Rivera A., Oridota O., Beauchamp W., Camacho-Rivera M. (2020). Social and demographic patterns of health related internet use among adults in the United States: A secondary data analysis of the Health Information National Trends survey. Int. J. Environ. Res. Public. Health.

[11] Govetto A., Virgili G., Rodriguez F.J., Figueroa M.S., Sarraf D., Hubschman J.P. (2019). Functional and anatomical significance of the ectopic inner foveal layers in eyes with idiopathic epiretinal membranes: Surgical results at 12 months. Retina.

[12] Massraf B., Chan K.K.C., Jain N., Panthagani J. (2025). Assessing accuracy, readability & reliability of AI-generated patient leaflets on Descemet membrane endothelial keratoplasty. Eur. J. Ophthalmol..

[13] Reyhan A.H., Mutaf Ç., Uzun I., Yüksekyayla F. (2024). A performance evaluation of large language models in keratoconus: A comparative study of ChatGPT-3.5, ChatGPT-4.0, Gemini, Copilot, Chatsonic, and Perplexity. J. Clin. Med..

[14] Thompson P., Thornton R., Ramsden C.M. (2025). Assessing chatbots ability to produce leaflets on cataract surgery: Bing AI, chatGPT 3.5, chatGPT 4o, ChatSonic, Google Bard, Perplexity, and Pi. J. Cataract. Refract. Surg..

[15] Güvenç U., Üney G., Ünlü N., Candan Ö., Orman G. (2025). Unveiling the limitations of OCT-based classification in diabetic epiretinal membranes: A call for integrative vascular and structural assessment with OCT-A. Int. Ophthalmol..

